# Comparative transcriptomics provides insight into the molecular basis of species diversification of section *Trigonopedia* (*Cypripedium*) on the Qinghai-Tibetan Plateau

**DOI:** 10.1038/s41598-018-30147-9

**Published:** 2018-08-03

**Authors:** Yan-Yan Guo, Yong-Qiang Zhang, Guo-Qiang Zhang, Lai-Qiang Huang, Zhong-Jian Liu

**Affiliations:** 1 College of Plant Protection, Henan Agricultural University, Zhengzhou, China; 2 Shenzhen Key Laboratory for Orchid Conservation and Utilization, The National Orchid Conservation Center of China and The Orchid Conservation and Research Center of Shenzhen, Shenzhen, China; 3 Center for Biotechnology and BioMedicine, Graduate School at Shenzhen, Tsinghua University, Shenzhen, China; 4 Center for Biotechnology and BioMedicine, Shenzhen Key Laboratory of Gene and Antibody Therapy, State Key Laboratory of Chemical Oncogenomics, Graduate School at Shenzhen, Tsinghua-Berkeley Shenzhen Institute (TBSI), Tsinghua University, Shenzhen, 518055 China; 5 Key Laboratory of National Forestry and Grassland Administration of Orchid Conservation and Utilization at College of Landscape Architecture, Fujian Agriculture and Forestry University, Fuzhou, China

## Abstract

Deceptive pollination is key to the species richness of Orchidaceae. However, the genetic basis of species diversification is still under study. Section *Trigonopedia* is a monophyletic clade of genus *Cypripedium* distributed in the southwest of China. The species of this section are pollinated by different flies. Pollinator differentiation makes section *Trigonopedia* an ideal group for studying the genetic basis underlying species diversification. Here, we sequenced the transcriptomes of eight species of the genus *Cypripedium*, including six co-flowering species of section *Trigonopedia* and two species outside this section as an outgroup. We reconstructed the phylogeny of the section with the combined 1572 single-copy genes extracted from the eight species and produced a highly resolved tree of the section. Furthermore, we combined substitution rate estimation and differential expression analysis to identify candidate genes, including genes related to floral scent synthesis and environmental adaptation, involved in species differentiation. Field investigations showed that these species have adapted to different habitats. We propose that the species diversification in this section is initiated by floral scent differentiation, followed by habitat differentiation, finally leading to speciation. This study sheds novel light on the diversification of closely related orchid species in the Qinghai-Tibetan region.

## Introduction

Speciation, which is important to ecology and evolution, is a key question puzzling orchid biologists. Orchidaceae, with a large variety of species, is the largest family of monocotyledons and provides valuable material for research on evolution. Approximately one-third of orchid species are pollinated by deceit, which is important to the species richness of Orchidaceae^1^. Pollinators are attracted by floral colour, reward, morphology, size, and scent. Among these attractants, floral scent plays an important role in pollinator attraction^2–4^. The interactions between pollinator behaviour and floral traits lead to the prezygotic reproductive isolation of plant taxa^5–7^. Existing studies of orchid deceptive pollination mainly focus on pollination biology and the evolution of sexually deceptive orchids; although progress has been made based on the sexually deceptive orchids^8–10^, little attention has been paid to the underlying genetic basis of other deceptive pollination strategies. The inner genetic basis of deceptive pollination is still not well understood for other species. Next-generation sequencing provides an opportunity to investigate this inner genetic basis and provide clues regarding the candidate genes and pathways involved in floral scent biosynthesis^11^. Thus, this speciation model should be investigated in more non-model species.

The high ornamental and commercial values of orchids have promoted the transcriptome sequencing of a number of orchids^8,12–29^. Sedeek *et al*.^8^ sequenced the transcriptomes and proteomes of three *Ophrys* species and identified candidate genes for pollinator attraction and reproductive isolation among sexually deceptive orchids. In addition, Orchidstra 2.0 collected 18 orchid transcriptomes, covering 12 genera in five subfamilies of Orchidaceae; these transcriptomes are important for understanding the genetic background of the biological process^26^. In addition, several diverse metabolic pathways are involved in floral scent synthesis, and the differential expression of candidate genes contributes to the odour difference^11^. Thus, additional studies should be carried out in closely related species.

The Qinghai-Tibetan Plateau is a hot spot in the study of speciation. Section *Trigonopedia* of *Cypripedium*, pollinated by different flies, is endemic to the Qinghai-Tibetan Plateau. Section *Trigonopedia* is a monophyly clade of genus *Cypripedium*^30^. Following the infrageneric classification of Cribb^31^, section *Trigonopedia* includes section *Sinopedilum* of the infrageneric classification of Perner^32^. The section consists of 11 species endemic to the southwest of China, including Yunnan, Sichuan, and Gansu Provinces, except for *Cypripedium lichiangense*, which has expanded to the north of Myanmar. Section *Trigonopedia* is ebracteate and has two leaves. Most species of genus *Cypripedium* are primarily pollinated by bees, whereas the species of this section are pollinated by flies. The floral presentation, colour, and scent also give clues to the fly pollination of this section^31^. Pollination studies have shown the pollinator differentiation of this section^33–36^. For example, *C*. *micranthum* and *C*. *sichuanense* are co-flowering sympatric species endemic to Sichuan, with the former pollinated by fruit flies and the latter pollinated by dung flies^36^. *C*. *fargesii* is pollinated by flat-footed flies and has a flavour of rotten leaves^35^. In addition, it is interesting that the predominant compound emitted by *C*. *bardolphianum* is ethyl acetate (70.4%) with a fruity wine flavour, whereas the compounds emitted by *C*. *micranthum* are dominated by pentyl ester-acetic acid (44.8%), 2-(2-methoxyethoxy)-ethanol (27.1%), and hydroxyacetic acid (15.3%)^37^.

Previous studies have shown that species of section *Trigonopedia* attract pollinators via distinct floral scents. The floral scent is the key factor promoting reproductive isolation and finally leads to species differentiation. However, the genetic basis underlying pollinator differentiation is still under study. Section *Trigonopedia* is distributed on the Qinghai-Tibetan Plateau, which is characterised by high mountains and deep valleys that provide diverse habitats for species differentiation. In addition, we observed habitat differentiation in the field. For instance, *C*. *bardolphianum* grows in dry, open forest, whereas *C*. *micranthum* and *C*. *sichuanense* grow in close forest. Thus, section *Trigonopedia* is a model system for studying the genomic background of species diversification.

The species of the section are closely related, co-flowering, morphologically similar, and pollinated by different species, providing an ideal system with which to investigate speciation on the Qinghai-Tibetan Plateau. In this study, we performed transcriptome sequencing to obtain massive protein-coding genes of the section and unravel the fundamental characteristics of these species. We first constructed the phylogeny of the section based on single-copy genes at the genomic level. Then, substitution rates were estimated in the six species. This approach allowed us to ascertain the positively selected genes possibly related to species diversification. Finally, we compared the differentially expressed genes (DEGs) between the flowers and leaves of each species to identify the candidate genes involved in floral scent differentiation. We aim to bridge the gap between pollination studies and species differentiation and unravel the molecular basis of the species diversification of this section. The study of diversification in this section will provide new insights into the speciation, ecological habits and evolution of Orchidaceae.

## Results

### Transcriptome Assembly

**Figure 1:**
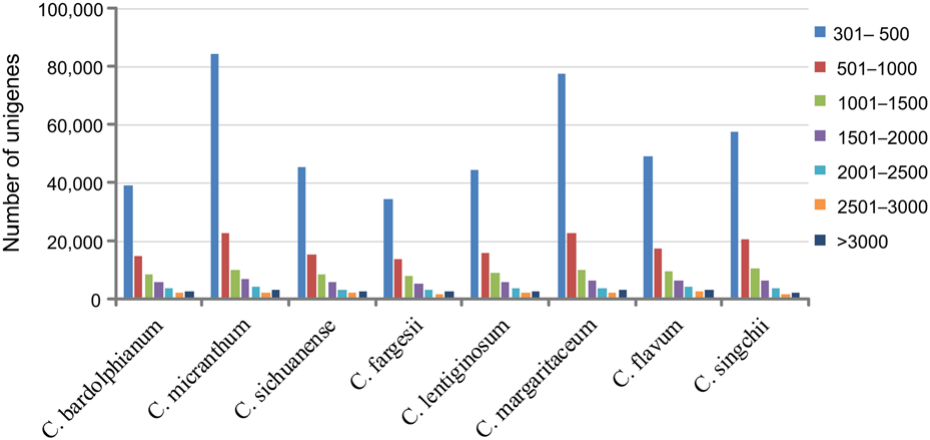
Sequence length distribution of unigenes.

In total, we obtained 15 transcriptome sequences representing eight species of genus *Cypripedium*, and each species had leaf and flower transcriptome sequences except *C. fargesii*, for which only the flower transcriptome was obtained (Table 1). After filtering adapter sequences and low-quality sequences, we obtained 33,748,574 (*C. sichuanense*, flower) to 69,005,058 (*C. micranthum*, leaf) clean reads (Table 1). After Trinity assembly, 68,870–133,597 unique transcripts were identified with a GC content of 42.77–44.76% and mean length of 583.89 bp–767.29 bp (Fig. 1, Table 1). Then, approximately 50% of the transcripts were filtered during transcription by TransDecoder. Finally, 32,949–45,037 proteins were translated from the assembly.

**Table 1:** Sources of materials and sequence assembly summary.

Taxa	Locations	Elevation (m)	Raw reads/Clean reads (million)	Transcripts/Unigenes	N50 of Transcripts/Unigenes
*C. bardolphianum*	Wanglang Nature Reserve	3100	52.6/50.7	76,907/55,871	1568/1409
*C. micranthum*	Huanglong Nature Reserve	2200	64.1/61.1	133,597/105,645	1244/875
*C. sichuanense*	Huanglong Nature Reserve	2200	44.9/41.9	83,247/62,869	1463/1257
*C. fargesii*	Jiuzhaigou Nature Reserve	2200	27.6/27.0	83,160/61,401	1496/1320
*C. lentiginosum*	Malipo, Yunnan	1800	45.6/43.2	68,870/51,458	1543/1374
*C. margaritaceum*	Zhongdian, Yunnan	3000	51.6/49.9	125,826/104,925	1244/1290
**Outgroup**					
*C*. *flavum*	Wanglang Nature Reserve	3100	49.4/47.8	92,941/70,822	1536/1268
*C*. *singchii*	Malipo, Yunnan	1800	34.0/29.6	102,819/75,059	1275/1084

### Phylogenetic Analyses of Section Trigonopedia

**Figure 2:**
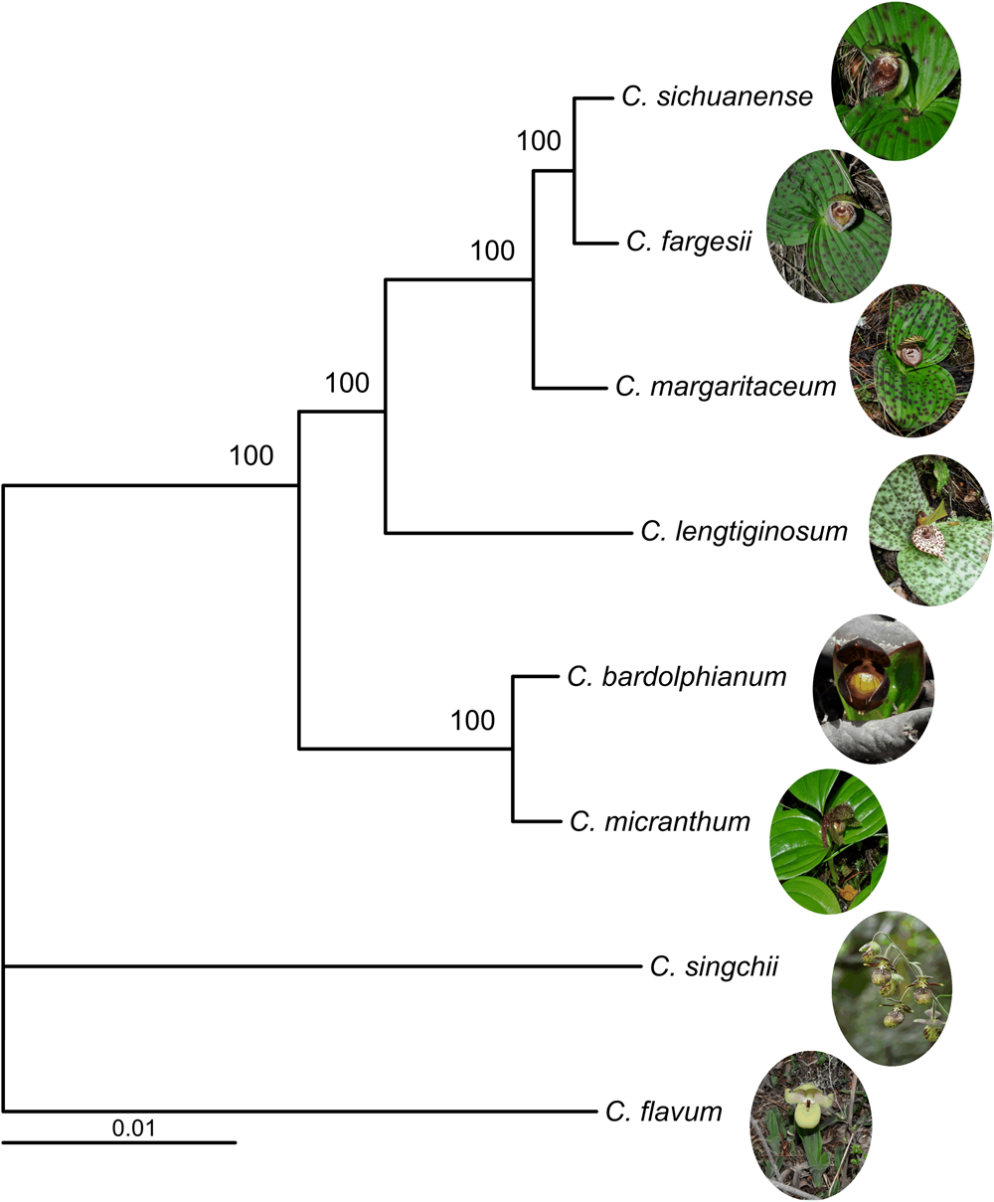
Phylogenetic tree of section *Trigonopedia* derived from 1572 concatenated single-copy genes.

A total of 1572 single-copy genes were identified from the eight sequenced species. The phylogenetic tree showed that the section was classified into two reciprocally monophyletic groups: one formed by *C. bardolphianum* and *C. micranthum* and the other formed by the other four species (*C. lentiginosum*, (*C. margaritaceum*, (*C. fargesii*, *C. sichuanense*)) (Fig. 2).

### Substitution Rate Estimation

**Figure 3:**
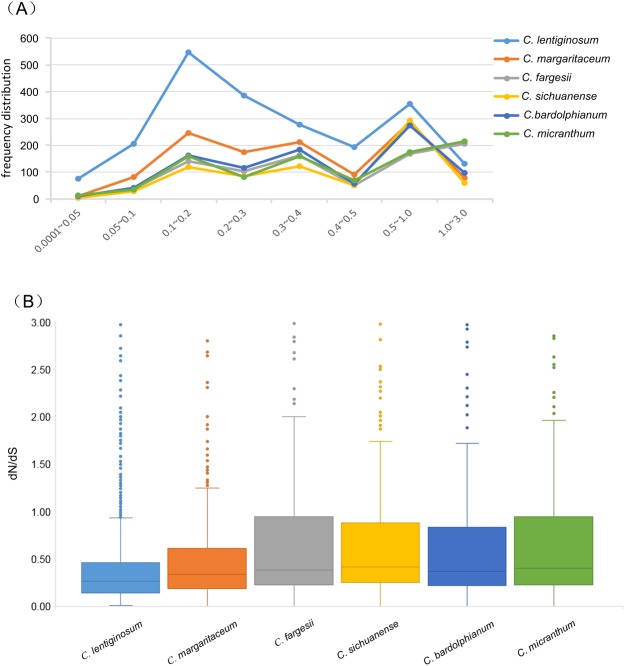
Number of orthologues with the given dN/dS ratios for the six species. (**A**) Frequency distribution of the dN/dS ratios. (**B**) The box plot shows the ratio of non-synonymous to synonymous mutations (dN/dS) for terminal branches estimated for each orthologue.

In the site model analysis, we detected footprints of positive selection in 67 genes, but only 8 of these genes could be KEGG annotated. Some of these genes may be associated with the diversification of this section (Table S1). One gene related to floral scent *(accB*), which takes part in several biosynthesis pathways, initiates the fatty acid biosynthesis. The other three genes were related to stress tolerance (*SOD2*, *rpoB*, and *MKS1*). In the branch model analysis, we detected 61 (*C. sichuanense*) to 216 (*C. micranthum*) positively selected genes in each lineage; *C. micranthum* (216) and *C. fargesii* (204) have more genes under positive selection than the other four species (61–131) (Fig. 3). The frequency distribution of dN/dS ratios showed that *C. lentiginosum* (485 genes) has more genes with elevated dN/dS ratios (3 > dN/dS > 0.5) than the other five species (354–389 genes) (Fig. 3). The boxplot results showed that *C. lentiginosum* had the lowest dN/dS median value (0.2649), while *C. sichuanense* had the highest median value (0.4207). The median values of the other four species were similar (Fig. 3). For each branch, most dN/dS ratios were in the range 0.5–1.0, except *C. lentiginosum* (Fig. 3). We also conducted KEGG functional classification for these PSGs in each branch. The distribution of KEGG classifications of PSGs included “Genetic Information Processing”, “Cellular Processes”, “Environmental Information Processing”, “Energy Metabolism”, “Lipid Metabolism”, “Metabolism of Cofactors and Vitamins”, “Carbohydrate Metabolism”, etc. “Genetic Information Processing” processes received the most hits in all the tested species. Each species has species-specific positive selected genes, especially genes with roles in floral scent (*accB*, *nadC*, *EPT1*, *GLYR, URH1*, and *FAB2*) and environmental adaptation. These genes may be closely related to the species differentiation in this section (Table S1); for example, *nad*C is involved in nicotinate and nicotinamide metabolism, and *FAB2* (K03921) is involved in fatty acid biosynthesis (Table S1). Most interestingly, we detected five genes (*PPIE*, *SNRPD3*, *HNRNPA1_3*, *SF3B2*, and *BCAS2*) involved in the spliceosome in *C. micranthum* (Table S1). In addition, both the site model and branch model indicated that *SOD2* was involved in the FOXO signalling pathway, which is related to stress tolerance.

### Differential Expression Analysis

**Figure 4:**
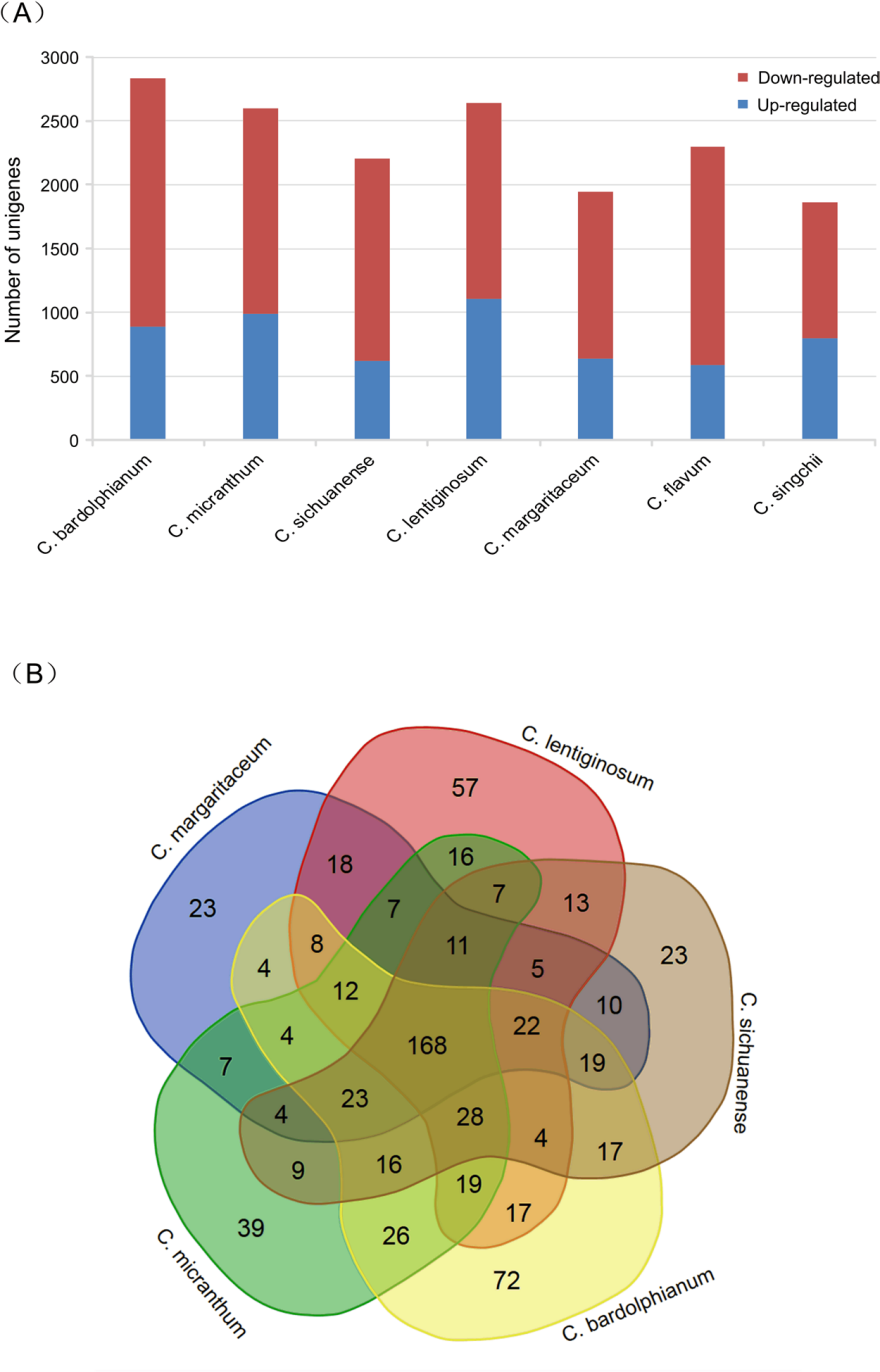
Comparison of the expression patterns of differential unigenes identified between flowers and leaves of section *Trigonopedia*. (**A**) Number of DEG unigenes between flowers and leaves. (**B**) Venn diagram showing unique and shared DEG unigenes.

Differential expression analysis between flowers and leaves indicated that *C*. *bardolphianum* has up to 2831 DEGs, followed by *C*. *lentiginosum* (2636), *C*. *micranthum* (2602), *C*. *sichuanense* (2207), and *C*. *margaritaceum* (1947), whereas 2294 DEGs (*C*. *flavum*) and 1860 DEGs (*C*. *singchii*) were found in the outgroup species. Species in section *Trigonopedia* have more up-regulated genes in leaves than in flowers (Fig. 4). The GO annotation of the differential expression genes was classified into three categories: biological process, molecular function, and cellular component (Fig. S1). The DEG enrichment analysis and Venn diagram both indicated that these species have section-shared DEGs and species-specific DEGs (Figs 4, S1). Five species of this section share DEGs related to fatty acid biosynthesis (*ACSL*); fatty acid degradation (*ACSL* and *MFP2*); ether lipid metabolism (*plc*); nicotinate and nicotinamide metabolism (URH1); diterpenoid biosynthesis (*GA3* and *E1.14.11.13*); and biosynthesis of terpenoids, steroids (*crtB*), etc., related to floral scent synthesis. Meanwhile, we identified *ispF* as being related to terpenoid backbone biosynthesis in *C*. *lentiginosum* and *ispD* as being related to terpenoid backbone biosynthesis in *C. bardolphianum*.

## Discussion

### The Phylogeny of Section *Trigonopedia*

Considering the tree of a single gene or few cpDNA/mtDNA cannot reveal the true relationships among species. Increasing studies use transcriptome data for phylogenetic construction^28,38–41^. Zhang *et al*.^28^ indicated that single copy genes were powerful tools for orchid phylogeny construction. Here, we used transcriptome methods to screen the genome-wide differentiation of closely related species. The phylogeny of the section, based on 1572 single-copy genes, shows that the section forms two reciprocally monophyletic groups with high support: the first clade formed by *C. bardolphianum* and *C. micranthum* and the other clade formed by the other four species (*C*. *lentiginosum*, (*C*. *margaritaceum*, (*C*. *fargesii*, *C. sichuanense*))) (Fig. 2). The species of the former clade have tiny flowers and no spots on the leaves, while the species of the other clade have relatively larger flowers and blackish purple spots on the leaves (Fig. 2). Our results are congruent with the cpDNA tree of previous studies and incongruent with the nrITS tree and concatenated gene tree (nrITS + cpDNA)^30^. The non-monophyly of the nonconcerted evolution of orthology and paralogy of nrITS may mislead the phylogenetic inference^42^, which may explain the inconsistency between this study and the nrITS gene tree. Moreover, the single-copy nuclear genes developed in this study could be beneficial for the phylogenetic construction of genus *Cypripedium*.

The classification of the two clades indicates the early diversification of these species. Species characterised by flowers without a bract below the ovary were gathered in section *Trigonopedia*^31^. Then, the species with tiny flowers and leaves were placed in section *Sinopedilum*, while the other species were placed in section *Trigonopedia*^32,43^. Our study supports the delimitation of two sections, which was also supported by the morphological characters. However, studies combining these species would further elucidate the evolution of the genus.

Species on the Qinghai-Tibetan Plateau are crucial for understanding species diversification in this region. The common ancestor of the section is dated to the Pliocene, and many species diversified in the Pleistocene^44^. Our results showed that neighbouring species are more closely related, e.g., *C. bardolphianum* and *C. micranthum*, with sister relationships grow in a sympatric distribution in Sichuan; *C. lentiginosum* is distributed in Yunnan at the base of the other clade, followed by *C. margaritaceum*; while *C. fargesii* and *C. sichuanense*, with overlapping distributions, form the sister clade, which agrees with the south to north niche shift of the genus^45^.

The field investigations indicated that the species of the genus have adapted to different habitats. For example, *C. bardolphianum* and *C. micranthum* are sister species but occupy different habitats; *C. bardolphianum* grows in the dry, open forest; and *C. micranthum* grows in the dense forest. The populations of *C. bardolphianum* and *C. sichuanense* are no more than 50 m apart in Danyunxia. *C. fargesii* may be moisture tolerant, and its population in Jiuzhaizhou is near a calcareous pool, which fills with water during the rainy period. However, the habitat of *C*. *margaritaceum* is dry in Zhongdian and Lijiang. Moreover, the altitude varies (Table 1). For example, *C. bardolphianum* and *C*. *margaritaceum* are found at 3000 m; *C. micranthum, C. sichuanense* and *C. fargesii* are found at 2200 m; and *C. lentiginosum* is found at 1800 m. Interestingly, species with a more restricted distribution also show more distinctive morphology, especially *C. lentiginosum*, which has larger flowers and longer leaves but is restricted to Malipo of Yunan, whereas *C. micranthum* is found only in Danyunxia of Huanglong. Species with peripheral distributions are strongly differentiated from other species. The frequency distribution of dN/dS ratios showed that *C. micranthum* has more genes under positive selection than the other five species (Fig. 2). The elevated dN/dS ratio of *C. lentiginosum* (485 genes) was higher than that of its relatives (354–389 genes), and more species-specific DEGs were found in *C. lentiginosum* than those in other species in this section (Fig. 4); these findings may explain the endemic distribution and morphology of *C. lentiginosum*. This habitat diversification may accelerate the inner genetic diversification in this section.

### Genes Related to Species Diversification of Section *Trigonopedia*

The positive selection and DEGs provide clues to the species differentiation of this section. Floral scents function as olfactory cues for pollinator attraction and play a vital role in reproductive isolation. Studies of sexually deceptive orchids have indicated that the interaction between floral scent and pollinators promotes speciation, e.g., *Chiloglottis*^46^ and *Ophrys*^3,47,48^. This phenomenon has also been discovered in other non-deceptive pollination groups, e.g., *Silene*^49^, *Petunia*^50^, and *Mimulus*^51^. Waelti *et al*.^49^ found that a difference in a single compound can affect the pollination efficiency. Previous studies have indicated that the metabolisation of floral scent compounds is related to multiple biosynthesis pathways, including the biosynthesis of terpenoids, phenylpropanoids/benzenoids, volatile fatty acid derivatives, and amino acid-derived volatiles^52^. Section *Trigonopedia* is pollinated by different species and exhibits a differentiation of floral scent composition^33–36^, which indicates that the floral scent-related genes are important for the species differentiation in this section.

We identified 12 candidate genes related to the biosynthesis of floral scent compounds: five were identified from selection analysis, and seven were identified from differential expression analysis. These genes cover nine gene pathways, e.g., fatty acid biosynthesis, fatty acid degradation, ether lipid metabolism, nicotinate and nicotinamide metabolism, diterpenoid biosynthesis, and biosynthesis of terpenoids and steroids (Tables S1, S2). These genes provide clues to the molecular basis of floral scent differentiation. The site model indicated that *accB* (dN/dS = 1.58077) has an elevated evolutionary rate. *accB* (the biotin carboxyl carrier) is the subunit of acetyl-CoA carboxylase, which initiates the synthesis of fatty acids. In addition, the branch model indicated that *FAB2* in *C. bardolphianum* (dN/dS = 1.0167) and *C. micranthum* (dN/dS = 1.1665) have an elevated evolutionary rate. The *FAB2* gene encoding acyl-[acyl-carrier-protein] desaturase is a homolog of SAD, which has an important role in the reproductive isolation in sexually deceptive orchids^53,54^. Sedeek *et al*.^54^ even proposed that *SAD5* is a speciation gene in *Ophrys*.

Our DEG findings indicate that species-specific and section-shared genes both contribute to the floral scent differentiation. The interplay between section-shared DEGs and species-specific DEGs may leads to the differentiation of floral scent. We identified seven section-shared candidate genes involved in the biosynthesis of floral scent compounds; for example, *ACSL* was a section-shared DEG involved in both fatty acid biosynthesis and fatty acid degradation, *MFP2* is related to fatty acid degradation, *GA3* and *E1.14.11.13* are related to diterpenoid biosynthesis, and *crtB* is related to the biosynthesis of terpenoids and steroids (Table S2). In addition, we identified species-specific DEGs; for example, we identified *ispF* as being related to terpenoid backbone biosynthesis in *C*. *lentiginosum* and *ispD* as being related to terpenoid backbone biosynthesis in *C. bardolphianum*. Due to the limitations of our study, there may be more species-specific DEGs involved in floral scent differentiation.

The other group of candidate genes involved in speciation is related to environmental adaptation. E.g. crassulacean acid metabolism might resulted from differential gene expression^28^. Our findings indicate that the “Environmental Information Processing” and “Energy Metabolism” genes in this section would be important for habitat differentiation. For example, *rpoB* encodes the RNA polymerase beta subunit, and its mutation causes extensive changes in gene expression in *Escherichia coli*^55^. We identified the positive selection of genes of the arachidonic acid metabolism pathway in *C. bardolphianum* (*LTA4H*) and *C. fargesii* (*gpx*); reportedly, this pathway is involved in desert adaptation in animals (Table 1). Interestingly, we detected seven positively selected genes involved in the spliceosome, five in *C. micranthum* (*PPIE*, *SNRPD3*, *HNRNPA1_3*, *SF3B2*, and *BCAS2*), four in *C. fargesii* (*SNRPD3*, *SF3B2*, *SF3A2*, and *TXNL4A*), and one in *C. lentiginosum* (*BCAS2*) (Table S1). Alternative splicing is closely associated with many abiotic stresses (salt stress, drought, flooding, temperature, etc.)^56^ and contributes to the evolution of phenotypic novelty^57^. We also detected genes related to DNA repair and recombination, including nucleotide excision repair and homologous recombination. Qiao *et al*.^58^ detected these genes in *Crucihimalaya himalaica*, with positive selection related to environmental adaptation on the Qinghai-Tibetan Plateau.

These genes involved in floral scent and environmental adaptation may be related to the species diversification of this section. This finding is compatible with field observations. The complex topography of the Qinghai-Tibetan Plateau provides diverse habitats, and some species of this section also inhabit differential habitats, which accelerates the speciation process of this genus. Floral scent pathway genes induced species differentiation, and then habitat adaptation accelerated the speciation of the section. Our findings provide insight into the role of floral scent in pollinator attraction, followed by adaptation to different habitats, and finally leading to speciation.

## Conclusions

This study provides valuable genomic resources for studying the molecular mechanism of closely related deceptively pollinated species with large and complex genomes. Our findings indicate that multiple genetic changes (positive selection and gene expression changes) may have contributed to the speciation process of this section and give clues to the molecular basis of floral scent differentiation. The Qinghai-Tibetan Plateau is the diversity centre of many species, and the differentiation of the species here is important for understanding species diversity in this region. Though a series of studies have been carried out, the genetic basis of species diversification is still under study. In this study, we investigated the diversification of section *Trigonopedia* through high-throughput sequencing and found candidate genes related to species diversification in this section through substitution rate analysis and differential expression analysis. Species diversification in this section involves the interplay between pollinator differentiation and habitat adaptation. Furthermore, the genetic basis of species differentiation provides new insights into orchid diversity and sheds light on the species diversification of orchids on the Qinghai-Tibetan Plateau.

## Materials and Methods

### RNA Extraction and Transcriptome Sequencing

We collected all the samples from fields in Sichuan and Yunnan, including six species (*C*. *bardolphianum*, *C*. *fargesii*, *C*. *lentiginosum*, *C*. *margaritaceum*, *C*. *micranthum*, and *C*. *sichuanense*) of section *Trigonopedia* and two species (*C*. *flavum* and *C*. *singchii*) outside this section that served as an outgroup. The detailed sample information is shown in Table 1. The RNA materials collected were stored in RNA*later* solution (Ambion) and preserved at −80 °C before RNA isolation.

Total RNA was isolated from leaves and flowers using a Tiangen^®^ RNAprep Pure Plant Kit (Tiangen, Beijing). The transcriptome library construction and Illumina sequencing were performed by Novogene (Beijing, China).

### Sequence Assembly, Functional Annotation, and Orthologue Identification

The raw reads were filtered to obtain high-quality clean reads by removing the adapter sequences or low-quality reads. The clean reads were deposited in the NCBI Short Read Archive (SRA) under the accession number SRP151832.

The clean reads were *de novo* assembled using Trinity (released 20140717)^59^, and contigs less than 300 bp were excluded. Cd-Hit-Est^60^ was used for a clustering analysis to reduce sequence redundancy. Then, the filtered sequences were translated with TransDecoder^61^. The translated amino acid sequences were annotated through blast to the PFAM database by InterProScan 5^62^. The annotated sequences were aligned with all-to-all blast by OrthoMCL^63^ with an e-value cut-off of 10^−5^ for gene family clustering. We constructed two databases: the first database comprised all eight species, and the other database was composed of the six ingroup species. The CDS sequences of each single-copy gene were aligned with MUSCLE^64^. Then, the alignments were manually refined in BioEdit 7.2.5^65^. Finally, 1572 single-copy genes remained for phylogenetic analyses and 2592 single-copy genes remained for substitution rate estimation.

### Phylogenetic Analyses

We screened single-copy genes for the phylogeny construction. We aligned each fragment with MUSCLE^64^ and concatenated the fragments for phylogeny construction. Then, we used RAxML-HPC BlackBox 8.2.10^66^ for the construction of the ML tree using the GTR + GAMMA model; this analysis was performed on the CIPRES Science Gateway V.3.3 platform^67^. We used the ML tree for the downstream analyses for positive selection.

### Substitution Rate Estimation

The CodeML programme implemented in PAML 4.9^68^ was used to estimate the non-synonymous substitution rate (dN) and synonymous substitution rate (dS) for each gene using the F3X4 codon model. The species tree ((*C. lentiginosum*, (*C. margaritaceum*, (*C. fargesii*, *C. sichuanense*))), (*C. bardolphianum*, *C. micranthum*)) was used as the guide tree. In addition, we estimated the dN/dS ratio of each branch using the free-ratio model (Model = 1) for each orthogroup. We conducted the boxplot analysis using the dN/dS values derived from the free-ratio model results and filtered for dN/dS > 3 or dN/dS < 0.0001. We also established frequency distribution plots of all the dN/dS ratios of the six species. To elucidate the biological functions of positive selected genes, we performed KEGG pathway analysis.

### Differential Expression Analysis, Annotation of DEGs, and Cluster Analysis

Gene expression levels were estimated with RSEM by assembling the clean reads of each sample to Trinity assemblies^69^. We then performed differential expression analysis using the edgeR package in R^70^. The identified DEGs were used for GO analysis. GO functional annotations were obtained for all DEGs using InterProScan 5^62^. GO enrichment analysis of the DEGs was performed using the WEGO online tool (http://wego.genomics.org.cn/)^71^. We then performed KEGG analysis for the DEGs.

## Electronic supplementary material

Fig. S1, Table S1, Table S2
