# Supplementary material for: Comparative transcriptomics provides insight into the molecular basis of species diversification of section Trigonopedia (Cypripedium) on the Qinghai-Tibetan Plateau
Source: Sci Rep. 2018 Aug 3;8:11640. doi: 10.1038/s41598-018-30147-9 (PMC6076244; doi:10.1038/s41598-018-30147-9)
Supplement: Supplementary file 1 — Fig. S1, Table S1, Table S2 [file 41598_2018_30147_MOESM1_ESM.pdf]

Supplementary material on

Comparative transcriptomics provides insight into the molecular basis of species diversification of section *Trigonopedia* (*Cypripedium*) on the Qinghai-Tibetan Plateau

Yan-Yan Guo[1, 2, 3], Yong-Qiang Zhang[2], Guo-Qiang Zhang[2], Lai-Qiang Huang[3, *], Zhong-Jian Liu[2, 4, *]

[1] College of Plant Protection, Henan Agricultural University, Zhengzhou, China

[2] Shenzhen Key Laboratory for Orchid Conservation and Utilization, The National Orchid Conservation Center of China and The Orchid Conservation and Research Center of Shenzhen, Shenzhen, China

[3] Center for Biotechnology and BioMedicine, Graduate School at Shenzhen, Tsinghua University, Shenzhen, China

[4] Key Laboratory of National Forestry and Grassland Administration of Orchid Conservation and Utilization at College of Landscape Architecture, Fujian Agriculture and Forestry University, Fuzhou, China.

Corresponding author:

Dr. Zhong-Jian Liu
The Orchid Conservation and Research Center of Shenzhen
No. 889, Wangtong Road, Shenzhen 518114, China
Fax: 86-755-25711928; E-mail: liuzj@sinicaorchid.org

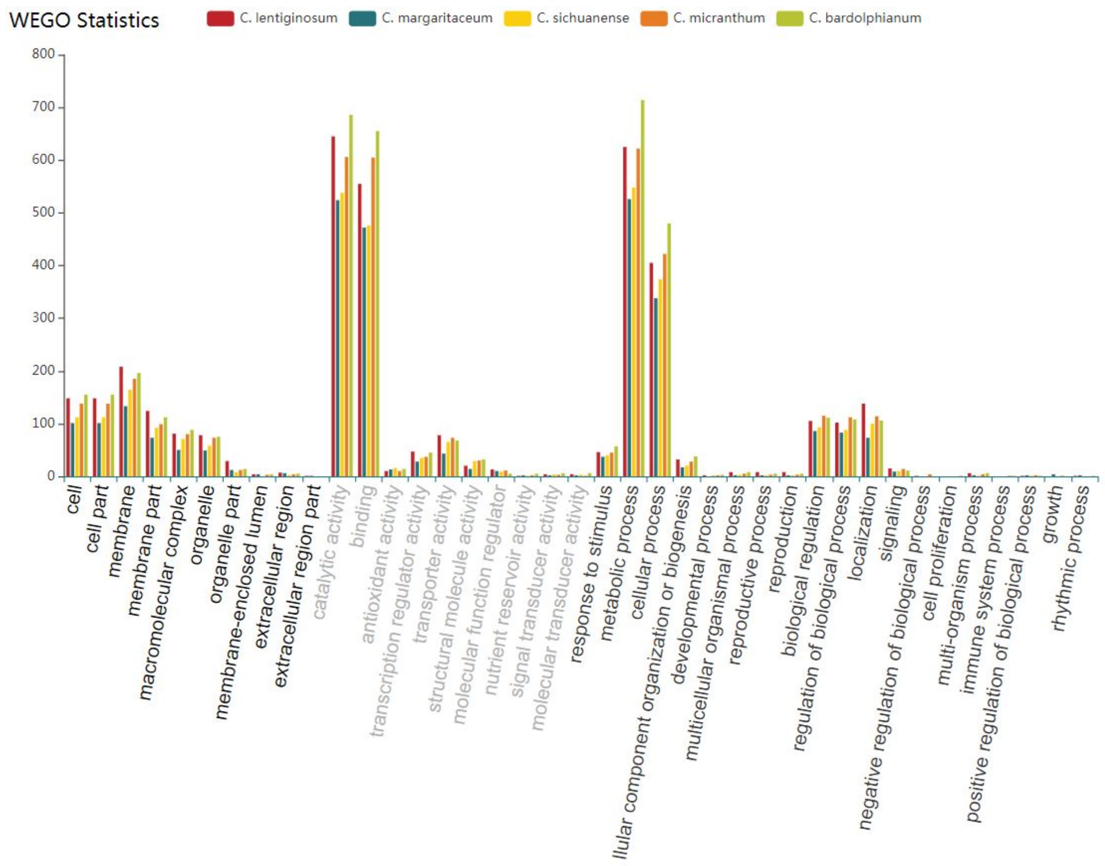

Fig. S1 GO term distributions of DEGs in section *Trigonopedia*.

Table S1 Candidate genes related to species differentiation in section *Trigonopedia* in selection analysis.

KEGG pathway	Gene	Definition	Ko id
**Site Model**			
Fatty acid biosynthesis	*accB*	acetyl-CoA carboxylase biotin carboxyl carrier protein	K02160
FOXO signalling pathway	*SOD2*	superoxide dismutase, Fe-Mn family	K04564
RNA polymerase	*rpoB*	DNA-directed RNA polymerase subunit beta	K03043
MAPK signalling pathway	*MKS1*	MAP kinase substrate 1	K20725
**Branch Model**			
C. margaritaceum			
Nicotinate and nicotinamide metabolism	*nadC*	nicotinate-nucleotide pyrophosphorylase (carboxylating)	k00767
base excision repair	*MBD4*	methyl-CpG-binding domain protein 4	K10801
MAPK signalling pathway	*EBF1_2*	EIN3-binding F-box protein	K14515
Necroptosis	*CHMP2A*	charged multivesicular body protein 2A	K12191
Plant hormone signal transduction	*EBF1_2*	EIN3-binding F-box protein	K14515
C. lentiginosum			
FOXO signalling pathway	*SOD2*	superoxide dismutase	K04564
Plant hormone signal transduction	*JAZ*	jasmonate ZIM domain-containing protein	K13464

Plant hormone signal transduction	*ARR-B*	two-component response regulator ARR-B family	K14491
Spliceosome	*BCAS2*	pre-mRNA-splicing factor SPF27	K12861
photosynthesis	*petJ*	cytochrome c6	K08906
photosynthesis	*psaH*	photosystem I subunit VI	K02695
photosynthesis	*ATPF0B*	F-type H+-transporting ATPase subunit b	K02109
Cellular senescence	*RBBP4*	histone-binding protein RBBP4	K10752
C. fargesii			
Ether lipid metabolism	*EPT1*	ethanolaminephosphotransferase	K00993
Butanoate metabolism	*GLYR*	glyoxylate/succinic semialdehyde reductase	K18121
Cutin, suberine and wax biosynthesis	*PXG*	peroxygenase	K17991
P53 signalling pathway	SIAH1	E3 ubiquitin-protein ligase SIAH1	K04506
Arachidonic acid metabolism	*gpx*	glutathione peroxidase	K00432
photosynthesis	*psaG*	photosystem I subunit V	K08905
photosynthesis	*psaN*	photosystem I subunit PsaN	K02701
photosynthesis	*psbO*	photosystem II oxygen-evolving enhancer protein 1	K02716
photosynthesis	*psbP*	photosystem II oxygen-evolving enhancer protein 2	K02717
photosynthesis	*petJ*	cytochrome c6	K08906

photosynthesis	*ATPF1E*	F-type H+-transporting ATPase subunit epsilon	K02114
Spliceosome	*SNRPD3*	small nuclear ribonucleoprotein D3	K11088
Spliceosome	*SF3B2*	splicing factor 3B subunit 2	K12829
Spliceosome	*SF3A2*	splicing factor 3A subunit 2	K12826
Spliceosome	*TXNL4A*	U5 snRNP protein, DIM1 family	K12859
Nucleotide excision repair	*XPC*	xeroderma pigmentosum group C-complementing protein	K10838
Homologous recombination	*RAD51*	DNA repair protein RAD51	K04482
MAPK signalling pathway	*MAPKKK17_18*	mitogen-activated protein kinase kinase kinase 17/18	K20716
C. sichuanense			
Nicotinate and nicotinamide metabolism	*URH1*	uridine nucleosidase	K01240
photosynthesis	*ATPF1E*	F-type H+-transporting ATPase subunit epsilon	K02114
C. bardolphianum			
Fatty acid biosynthesis	*FAB2*	acyl-[acyl-carrier-protein] desaturase	K03921
photosynthesis	*ATPF1E*	F-type H+-transporting ATPase subunit epsilon	K02114
Plant-pathogen interaction	*NOA1*	nitric-oxide synthase, plant	K13427
Arachidonic acid metabolism	LTA4H	leukotriene-A4 hydrolase	K01254
Necroptosis	*H2A*	histone H2A	K11251

C. micranthum			
Fatty acid biosynthesis	*FAB2*	acyl-[acyl-carrier-protein] desaturase	K03921
Pantothenate and CoA biosynthesis	*ilvD*	dihydroxy-acid dehydratase	K01687
Pantothenate and CoA biosynthesis	*coaW*	type II pantothenate kinase	K09680
Homologous recombination	*RAD51*	DNA repair protein RAD51	K04482
MAPK signalling pathway	*MAPKKK17_18*	mitogen-activated protein kinase kinase kinase 17/18	K20716
P53 signalling pathway	*SIAH1*	E3 ubiquitin-protein ligase SIAH1	K04506
Necroptosis	*H2A*	histone H2A	K11251
Necroptosis	*VPS4*	vacuolar protein-sorting-associated protein 4	K12196
Spliceosome	*PPIE*	peptidyl-prolyl isomerase E (cyclophilin E)	K09564
Spliceosome	*SNRPD3*	small nuclear ribonucleoprotein D3	K11088
Spliceosome	*HNRNPA1_3*	heterogeneous nuclear ribonucleoprotein A1/A3	K12741
Spliceosome	*SF3B2*	splicing factor 3B subunit 2	K12829
Spliceosome	*BCAS2*	pre-mRNA-splicing factor SPF27	K12861
photosynthesis	*psbR*	photosystem II 10 kDa protein	K03541
photosynthesis	*petJ*	cytochrome c6	K08906
photosynthesis	*ATPF1E*	F-type H+-transporting ATPase subunit epsilon	K02114

Table S2 Candidate genes related to floral scent synthesis in section *Trigonopedia* in differential expression analysis.

KEGG pathway	Gene	Definition	Ko id
**Section-shared genes**			
Fatty acid biosynthesis	*ACSL*	long-chain acyl-CoA synthetase	K01897
Fatty acid degradation	*ACSL*	long-chain acyl-CoA synthetase	K01897
Fatty acid degradation	*MFP2*	enoyl-CoA hydratase/3-hydroxyacyl-CoA dehydrogenase	K10527
Ether lipid metabolism	*plc*	phospholipase C	K01114
Nicotinate and nicotinamide metabolism	*surE*	5'-nucleotidase	K03787
Diterpenoid biosynthesis	*GA3*	ent-kaurene oxidase	K04122
Diterpenoid biosynthesis	*E1.14.11.13*	gibberellin 2-oxidase	K04125
Biosynthesis of terpenoids and steroids	*crtB*	15-cis-phytoene/all-trans-phytoene synthase	K02291
Species-specific genes			
C. lentiginosum			
Pyruvate metabolism	*E4.2.1.2B*	fumarate hydratase, class II	K01679
Nicotinate and nicotinamide metabolism	*ppnK*	NAD+ kinase	K00858
Terpenoid backbone biosynthesis	*ispF*	2-C-methyl-D-erythritol 2,4-cyclodiphosphate synthase	K01770
C. bardolphianum			

Terpenoid backbone biosynthesis	*ispD*	2-C-methyl-D-erythritol 4-phosphate cytidylyltransferase	K00991
Fatty acid biosynthesis	*ACSL*	long-chain acyl-CoA synthetase	K01897
